# Antimicrobial Activity and Probable Mechanisms of Action of Medicinal Plants of Kenya: *Withania somnifera, Warbugia ugandensis, Prunus africana* and *Plectrunthus barbatus*

**DOI:** 10.1371/journal.pone.0065619

**Published:** 2013-06-13

**Authors:** Peter G. Mwitari, Peter A. Ayeka, Joyce Ondicho, Esther N. Matu, Christine C. Bii

**Affiliations:** 1 Center for Traditional Medicine and Drug Research, Kenya Medical Research Institute, Nairobi, Kenya; 2 Tianjin University of Traditional Chinese Medicine, Tianjin, PR China; 3 Egerton University, Egerton, Kenya; 4 Center for Microbiology Research, Kenya Medical Research Institute, Nairobi, Kenya; Beijing Institute of Microbiology and Epidemiology, China

## Abstract

*Withania somnifera, Warbugia ugandensis, Prunus africana* and *Plectrunthus barbatus* are used traditionally in Kenya for treatment of microbial infections and cancer. Information on their use is available, but scientific data on their bioactivity, safety and mechanisms of action is still scanty. A study was conducted on the effect of organic extracts of these plants on both bacterial and fungal strains, and their mechanisms of action. Extracts were evaluated through the disc diffusion assay. Bacteria and yeast test strains were cultured on Mueller-Hinton agar and on Sabouraud dextrose agar for the filamentous fungi. A 0.5 McFarland standard suspension was prepared. Sterile paper discs 6 mm in diameter impregnated with 10 µl of the test extract (100 mg/ml) were aseptically placed onto the surface of the inoculated media. Chloramphenicol (30 µg) and fluconazole (25 µg) were used as standards. Discs impregnated with dissolution medium were used as controls. Activity of the extracts was expressed according to zone of inhibition diameter. MIC was determined at 0.78–100 mg/ml. Safety studies were carried using Cell Counting Kit 8 cell proliferation assay protocol. To evaluate extracts mechanisms of action, IEC-6 cells and RT-PCR technique was employed *in vitro* to evaluate Interleukin 7 cytokine. Investigated plants extracts have both bactericidal and fungicidal activity. *W. ugandensis* is cytotoxic at IC_50_<50 µg/ml with MIC values of less than 0.78 mg/ml. *Prunus africana* shuts down expression of IL 7 mRNA at 50 µg/ml. *W. somnifera* has the best antimicrobial (1.5625 mg/ml), immunopotentiation (2 times IL 7 mRNA expression) and safety level (IC_50_>200 µg/ml). Fractions from *W. ugandensis* and *W. somnifera* too demonstrated antimicrobial activity. Mechanisms of action can largely be attributed to cytotoxicity, Gene silencing and immunopotentiation. Use of medicinal plants in traditional medicine has been justified and possible mechanisms of action demonstrated. Studies to isolate and characterize the bioactive constituents continue.

## Introduction

Traditional medicine is the main source of medical care for a great proportion of the population of the developing world. In Africa, indigenous plants play an important role in the treatment of a variety of diseases [1]. WHO (1996) has listed 21,000 plants that have medicinal uses around the world [2]. Plants Resources of Tropical Africa [3] has documented 2,200 priority medicinal plants in Tropical Africa. Kenya is rich in medicinal plants useful in treatment of common ailments as well as chronic diseases.

Microbial infections such as tuberculosis, candidiasis, cryptococcosis and salmonellosis are some of the infections that have been on the increase in the recent past partly due to HIV/AIDS pandemic. Resistance to anti-biotics such as norfloxacin, ciprofloxacin and amoxicillin-clavulanic acid by *Pseudomonas aeruginosa* and enterohemorrhargic *Escherichia coli* has been noted [4]. Multi-drug resistance poses serious challenges to the medical field and infections caused by multi-resistant bacteria especially in the intensive care units pose a huge problem [5].

Use of plant products for the control of human diseases has certain advantages besides being cheap to produce; they are biodegradable and readily available. Effective plant extracts can combat human pathogenic bacteria without toxic side effects and environmental hazards [6]. There is renewed interest in the search for plants with anti-microbial activity leading to various plants including *Azandirachta indica, Camelia sinensis, hypericum perforatum, Allium sativum* among others being investigated and, they displayed considerable antibacterial activity [6]. The analysis of the oil of *Rynchosia minima* shows that it contains β-caryophyllene (30.4%), gemacrene B (17.9%), camphor (7.8%), α-humulene (7.4%) and γ-muurolene (7.3%). The oil has shown significant inhibition against *B. cereus, S. aureus and M. luteus* [7]. *Rosmarinus officinalis, Salvia officinalis, Cinnamomum cassia* and *Syzygium aromaticum* have been examined against *P. aeruginosa* with *S. aromaticum* methanolic extract showing high inhibition activity against the bacterial strain resistant to several antibiotics including ampicillin and erythromycin [4]. The essential oils of *Piper nigrum*, *Szygyium aromaticum, Pelargonium graveolens* all from varying plant families show inhibition against both gram positive and gram negative bacteria [8]. *Warbugia ugandensis* Sprague is highly esteemed for its valuable pharmaceutical properties and is rated as second highest priority medicinal plant species in Kenya for detailed study [9], [10]. Warburganal and muzigadial from *W. ugandensis* exhibit very potent antifungal, antiyeast and plant-growth regulating activity [11]. Dried bark is commonly chewed and the juice swallowed as a remedy for stomach-ache, constipation, toothache, cough, fever, muscle pains, weak joints and general body pains [10], [12], [13], [14]. Fresh roots are boiled and mixed with soup for the prevention of diarrhoea. *Prunus africana* (Hoolh f.) Kalkm is a useful timber tree and the bark is used for liver problems and constipation [13]. Extracts of *P. africana* have been shown to have antibacterial and antifungal activity [15]. *Plectranthus barbatus*, more commonly known as *Coleus forskohlii* or Indian Coleus (In Kikuyu: *Maigoya*), is a tropical perennial plant. One of the most studied Plectranthus-derived compounds is the labdane forskolin isolated from *Plectranthus barbatus*. It has a range of pharmacological properties and could explain many of the diverse medicinal uses of *Plectranthus barbatus* [16]. In Kenya it is also referred to as *Kikuyu toilet paper*, as in rural areas its leaves are used as such. In India leaves and roots of *Plectranthus barbatus* have been traditional remedies in India for digestive complaints, heart and lung conditions, asthma, insomnia, muscle spasm, convulsions and skin disease [17]. *Withania somnifera* (L.) Dunal contains more than 80 chemical compounds, mainly alkaloids and steroids (withanolides). Numerous studies have been published on the activities of these compounds, mostly obtained from the leaves and roots. These studies have demonstrated antibiotic, anti-inflammatory, cytotoxic, anti-tumor and cholesterol-lowering activities [18], [19]. This is an important plant in the traditional medicine of Africa and Asia. The chemistry of *W. somnifera* has been extensively studied and over 35 chemical constituents have been identified, extracted, and isolated. The biologically active chemical constituents are alkaloids (isopelletierine, anaferine), steroidal lactones (withanolides, withaferins), saponins containing an additional acyl group (sitoindoside VII and VIII), and withanolides with a glucose at carbon 27 (sitoindoside IX and X). *W. somnifera* is also rich in iron [19], [20].

It is justifiable to search for alternative therapy in natural products, as plants have been known for many years as a source of therapeutic agents. Few researches have correlated *in vitro* activity, safety studies and mode of action besides isolating the bioactive compound(s). Different kinds of studies on the mechanisms of action should be given high priority [21]. This study sought to study activity, safety and identify the mode of action of the plant extracts of medicinal plants used in Traditional medicine to better develop safe drugs targeting their site of action.

Intestinal epithelial cells (IEC) have been implicated in IL-7 synthesis [22]. IL-7 plays an important role in immune processes in our bodies. Several studies have indicated that IEC may play an important role in mucosal immune responses by helping to regulate intestinal intraepithelial lymphocytes (IEL) [23]. Importance and usefulness of Cytokine IL 7 as a tool in immunologic activities has been demonstrated before [24]. In the current study, it is hypothesized that plant extracts act on the intestinal mucosal cells (IEC) which results in up regulation of the production of IL-7. IL-7 then elicits favorable conditions for other factors to come into play boosting immune response to microbial infections. IL-7 has a potential for adoptive immunotherapy [25], [26]. Drug agents that enhance or stimulate the production of IL-7 therefore provide potential candidates in microbial treatment as immune boosters. IL-7 is thus a viable research tool in evaluation of potential plant medicines and their mode of action. Immunological implications were demonstrated using IEC-6 cells.

The WST-8 cell quantitation kit from Dojindo is an ideal solution to the rapid determination of cell numbers for cell proliferation or cytotoxicity studies. The kit provides a single ready to use reagent that can be added directly to the cell cultures, without the need to harvest or wash the cells. The end product is highly soluble in aqueous solutions, is non-toxic and does not require solubilization prior to measuring the absorbance [27]. Application of this kit achieved desired results.

In this study, the medicinal plants; *Withania somnifera*, *Prunus Africana, Warbugia ugandensis* and *Plectranthus barbatus*used traditionally as sources of medicine were investigated bearing in mind that gaps exist in knowledge either in their bioactivity, safety and mode of action. They were collected from their natural environment in Ngong forest and sorrounding areas in Kenya. The plant extracts were evaluated for their antimicrobial activity, safety and mode of action. Their antimicrobial activity and mechanisms of action were demonstrated.

## Methods

### Approval, Collection and Extraction of Medicinal Plant Materials

Approval to carry out this research was given by the Kenya Medical Research Institute’s Scientific Steering Committee through research project number SSC. No. 1314.

Verbal permission was sought from the Kenya Wildlife Service (KWS) site office to collect stem bark of W.ugandensis & *P. africana* species from the road reserve adjacent to the Ngong forest. Stem barks were harvested in a non destructive way (without ringing the bark). Only small amount for purposes of research were obtained. Collection was done in company of Mr. Mutiso of the botany Department University of Nairobi and with close supervision by KWS rangers. *W. somnifera* was collected from abandoned Masai homesteads (Bomas) where it grows as a weed. *Plectranthus barbatus* also treated as a weed or sometimes established along fences in homesteads and no permission was required to pick a few leaves. These latter two species are not listed as endangered or protected species.

The stem barks for *W. ugandensis, P. africana* and aerial parts of *W. somnifera* and *P. barbatus* collected from their natural environment were identified by Mutiso of Botany department University of Nairobi. Voucher specimens are stored at the University of Nairobi Herbarium. Dried plant materials were ground using a laboratory mill; 50 g of each was weighed and put in a flat-bottomed conical flask, solvent added to cover the plant material completely and left to stand for 24 hours. Filtration was done and more solvent added and left to stand for a further 24hoursfollowed by filtration. The accrued filtrate was dried using a rotary evaporator, weight was taken, recorded and the extract stored in a cool dry place. Sequential extraction with hexane, dichloromethane, ethyl acetate and methanol was employed and resulting extracts employed in antimicrobial studies. Active extracts were fractionated for further antimicrobial evaluation. Isolation and purification of pure compounds was done through silica gel column.

Alternatively, dried plant materials were ground using a laboratory mill; 200 g of each was cold macerated in 300 ml of 95–75% ethanol for 24 hours and filtered. Re-extraction with 200 ml was done for a further 24hours. The accrued filtrate was dried using a rotary evaporator, weight was taken, recorded and the extract stored at 4°C until use. This procedure was repeated for all the Medicinal plant materials under investigation. These total extracts were used to carry out studies using Mouse IEC-6 cells.

### Antimicrobial Bio-assay

Antimicrobial activity was carried out using disc diffusion method [28], [29], [30]. Antibacterial activity was done on Mueller Hinton agar (Oxoid) using *Staphylococcus aureus* ATCC 25923, clinical isolate Methicilin Resistance *Staphylococcus aureus, Escherichia coli* ATCC 25922 *and Pseudomonas aeruginosa ATCC 27853*. Bacteria were maintained at 4°C on nutrient agar (NA) plates. Antifungal activity was determined on sabourauds dextrose agar (Oxoid) using *Candida albicans* ATCC 90028 and clinical isolates of *Cryptococcus neoformans*, *Microsporum gypseum*, and *Trichophyton mentagrophytes*. Bacterial and fungal strains used were acquired by and stored at the Centre for Microbiology Research, Kenya Medical Research Institute. Bacteria and yeast test strains were cultured on Mueller-Hinton agar for 24 hrs at 37°C and 35°C respectively and on Sabouraud dextrose agar at 30°C for 72 hrs for the filamentous fungi. A 0.5 McFarland standard suspension was prepared in normal saline. For filamentous fungi, mycelia suspension was used. The suspension was spread uniformly on Muller Hinton agar for bacteria and sabouraud dextrose agar (SDA) for fungal strains. A Whatman No. 3 sterile paper disc 6 mm in diameter was impregnated with 10 µl (100 mg/ml) of the test extracts, dried in a clean bench before aseptically placing onto the surface of the inoculated media. The plates were then incubated at temperatures of 35°C for yeast, 37°C for bacteria for 24 hrs and, 30°C for filamentous fungi for 72 hrs. The zones of inhibition were measured as indicators of activities. All the tests were done in triplicate. Chloramphenicol (30 µg) and fluconazole (25 µg) were used as standards. Discs impregnated with extraction solvents were used as controls [30].

The diameter of inhibition zone around each disc was measured and recorded at the end of incubation period. The average of the triplicate tests was taken. The degree of activity of the extracts was expressed according to inhibition zone diameter as follows; no activity (<7 mm), 8–11 mm active, >12 mm very active.

### Determination of Minimum Inhibitory Concentration (MIC)

The MIC was determined by impregnating paper discs with 10 µl of the reconstituted samples at a concentration ranging from 0.78–100 mg/ml. The discs were then transferred aseptically into Mueler Hinton agar plates (bacteria) or Sabouraud’s Dextrose Agar plates (fungi) inoculated with the test organisms. The MIC was regarded as the lowest concentration that produced a visible zone of inhibition [31].

### IEC-6 Cells Proliferation Assay

IEC-6 cells [32] (ATCC) were seeded at 50,000 cells per well in corning 96 well flat bottomed micro titer plates and incubated overnight at a volume of 100 µl. 10 µl plant extracts (*W. ugandensis, W. somnifera, P. africana & P.barbatus*) at eight different concentrations each serially diluted were added and cells incubated for a further 72hours. 10 µl of CCK-8 (Dojindo Molecular Technologies, Beijing, China) was then added and incubated in a high humidity environment at 37°C and 5% CO_2_ for 3 hours and optical difference (OD) read at 460 nm in a 96-well microtiter plate Tecan i- control infinite 200 OD reader. The test was done in triplicate. The data was analyzed using unpaired Student’s *T*-test.

### RNA Extraction, Amplification and Gel Electrophoresis

Extraction and amplification was done according RNeasy® Mini Kit (Qiagen).

The IEC-6 cells (ATCC) were cultured in 6 well plates in media supplemented with various plant extracts at appropriate concentrations for 72 hours. Briefly the media was decanted and cells washed in D-hanks solution and 1 ml Trizol (Invitrogen) added. 0.2 ml of Chloroform at 4°C was added per 1 ml shaken vigorously using a vortex for 30 seconds and incubated at room temperature (about 20°C) for a period of 4 minutes. This was followed by centrifuging for 15 minutes at 12000 revolutions per minute (rpm). A colorless aqueous layer formed at the top of which 500 µl was pipetted out carefully. A similar volume (500 µl) of isopropyl alcohol at minus 20°C was added to the RNA fraction and vortexed properly and incubated at room temperature (about 20°C) for a period of 25 minutes. The mixture was centrifuging for 10 minutes at 12000 revolutions per minute (rpm) at 4°C. The supernatant was discarded and RNA pellet washed with 1 ml of 75% ethanol and vortexed properly and, centrifuging for 10 minutes at 8000 revolutions per minute (rpm) at 4°. A refrigerated centrifuge was used each time. The supernatant was discarded and RNA pellet air dried before re-dissolving in 20 µl RNAse free water. The Optical difference (OD) was measured at 260/280 nm to determine the quality of RNA using a NanoDrop ND-1000 spectrophotometer (NanoDrop Technologies, Inc., Wilmington, DE, USA) and concentration (ng/µl) obtained. The concentration of RNA (ng/µl) was used to calculate the volumes of RNA and water (H_2_O) for use in reverse transcription by first dividing 500 by ng/µl of RNA obtained to give volume of RNA in µl and then this was subtracted from 6.5 to give volume of H_2_O to be used. Reverse transcription and cDNA amplification was done according to RNeasy® Mini Kit (Qiagen), and the following genes were targeted for amplification; Glyceraldehyde 3-phosphate dehydrogenase (GAPDH) with primers sequence 5′ to 3′ sense ACC ACA gTC CAT gCC ATC AC and antisense TCC ACC ACC CTg TTg CTg TA and, Interleukin 7 (IL-7) sense gAg TTT CAg ACg gCA CAC AA and antisense gAA ACT TCT ggg Agg gTT CC (from Sangon co.ltd) at reaction conditions (94°C for 3 minutes, 94°C for 30 seconds, 60.5°C for 30 seconds, final extension was at 72°C for 30 seconds) and at 22 cycles for GAPDH and 38 cycles for IL-7. The primers were designed using primer 3 [33]. Product was detected by agarose gel electrophoresis. All amplified cDNA were analyzed by 1.5% agarose gel electrophoresis and stained with ethidium-bromide for visualization under UV illumination (GeneGenius) and photographed. The size of amplified DNA was identified by comparison with DNA marker (100 bp DNA ladder, TaKaRa Biotechnology (Dalian) co., Ltd). Volumes of the DNA were calculated for each band. GAPDH served as an internal control.

### Data Analysis

Statistical analysis was done using excel data sheets and Statview version 5.0.1. The expression of IL-7 mRNA relative to GAPDH mRNA was calculated and, tables and bar charts drawn. The differences between the control and the treatments in these experiments were tested for statistical significance by unpaired Student’s *t*-test. A value of *p*≤0.05 was considered to indicate statistical significance. Values are expressed as mean±S.D.

## Results

### Antimicrobial Activities

**Figure 1:**
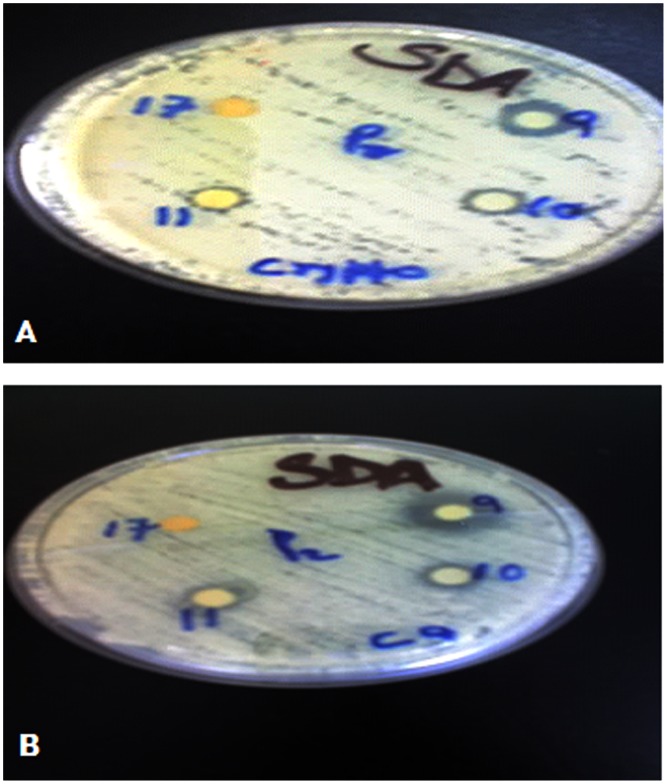
Antifungal activity of fractions 9, 10, 11 & 17 from dichloromethane extract of W. ugandensis against C. neoformans (A) and C. albicans (B). They display zones of inhibition (mm) of 12, 9, 8, 6 & 14, 8, 8, 6 respectively.

The ground plant medicines were subjected to sequential extraction to yield a hexane, dichloromethane, ethyl acetate and methanol part. The results of the extracts which showed good activity are shown in table 1. The dichloromethane and ethyl acetate parts of *W. somnifera* (WS) was very active against *Staphylococcus aureus* (SA) and Methicilin Resistance *Staphylococcus aureus* (MRSA), the methanol and ethyl acetate parts of *W. ugandensis* (WU) were also very active with the dichloromethane part being moderately active against SA and MRSA, The methanol part of *P africana* (PA) was very active with the ethyl acetate part being moderately active against SA and MRSA. WS dichloromethane and ethyl acetate parts show some activity against *Microsporum gypseum* (MG), and *Trichophyton mentagrophytes* (TM) but no activity against *Candida albicans* (CA) and *Cryptococcus neoformans* (CN) The dichloromethane and ethyl acetate parts of WU on the other hand are moderate to very active against *Microsporum gypseum*, *Candida albicans* and *Cryptococcus neoformans*. The MIC’s of the extracts show that dichloromethane and ethyl acetate parts of WU are much lower than 0.78 mg/ml the lowest concentration evaluated across the board. Most of the plant extracts and fractions evaluated have MIC’s of 12.5 mg/ml or lower (Table 2). On fractionating the Dichloromethane part of *W. ugandensis*, fractions 9, 10 & 11 showed good antifungal activity (Figure 1).

**Table 1:** Zones of inhibition (mm) of selected plant extracts against bacterial and fungal strains.

Extract/Microbe		SA	MRSA	MG	CA	CN	TM
Control	DMSO	6	6	6	6	6	6
	Chloramphenicol	18	24	NA	NA	NA	NA
	Fluconazole	NA	NA	15	15	15	15
Dichloromethane	WS	16	14	10	7	6	12
	WU	10	9	11	12	20	ND
Ethyl acetate	WS	14	12	8	6	6	8
	WU	13	13	13	13	22	ND
	PA	10	12	ND	ND	ND	ND
Methanol	WU	15	14	ND	ND	ND	ND
	PA	13	14	ND	ND	ND	ND

WS (W. somnifera), WU (W. ugandensis), PA (P. africana), SA (Staphylococcus aureus), MRSA (Methicilin Resistance Staphylococcus aureus), MG (Microsporum gypseum), CA (Candida albicans), CN (Cryptococcus neoformans), TM (Trichophyton mentagrophytes), NA (Not applicable), ND (Not done).

**Table 2:** The MIC’s in mm of selected extracts against bacterial and fungal strains.

Microbe/plant extract		SA	MRSA	PSDO	E. Coli	CA	CN	MG	TM
Dichloromethane	WS	6.25mg/ml	12.5mg/ml	12.5mg/ml	ND	ND	ND	ND	3.125mg/ml
	WU	3.125mg/ml	3.125mg/ml	ND	ND	<0.78mg/ml	<0.78mg/ml	<0.78mg/ml	ND
	PA	ND	ND	25mg/ml	ND	ND	ND	ND	ND
Ethyl acetate	WS	6.25mg/ml	12.5mg/ml	12.5mg/ml	ND	ND	ND	ND	1.5625mg/ml
	WU	0.78mg/ml	<0.78mg/ml	ND	12.5mg/ml	<0.78mg/ml	<0.78mg/ml	<0.78mg/ml	ND
	PA	12.5mg/ml	25mg/ml	ND	ND	ND	ND	ND	ND
Methanol	WU	6.25mg/ml	6.25mg/ml	ND	ND	ND	ND	ND	ND
	PA	0.78mg/ml	3.125mg/ml	ND	ND	ND	ND	ND	ND

WS (W. somnifera), WU (W. ugandensis), PA (P. africana), SA (Staphylococcus aureus), MRSA (Methicilin Resistance Staphylococcus aureus), MG (Microsporum gypseum), CA (Candida albicans), CN (Cryptococcus neoformans), TM (Trichophyton mentagrophytes), ND (Not done).

### Cell Proliferation

**Figure 2:**
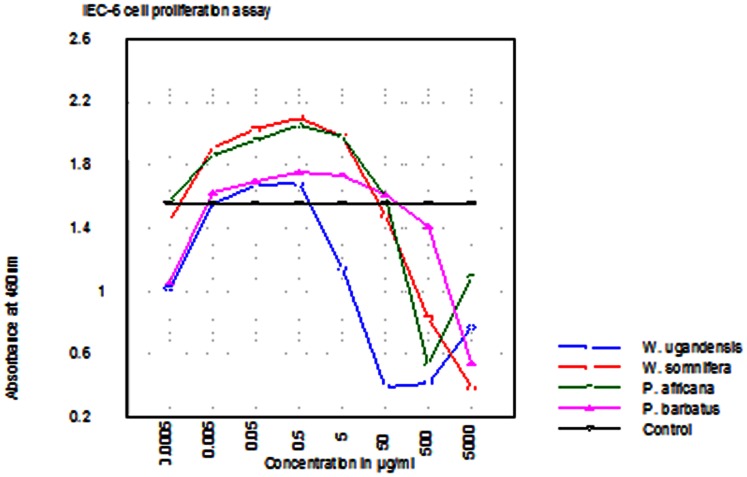
Graphical representation of the results of IEC-6 cell proliferation/cytotoxicity assay of 4 plant extracts. W.ugandesis (Wu), P. africana (Pa) & P. barbatus (Pb) at concentrations ranging from 0.0005–5000 µg/ml and, W. somnifera (Ws) at concentrations ranging from 0.001–10,000 µg/ml. W. somnifera (Ws), P. africana (Pa) & P. barbatus (Pb) are relatively safe for use even in dose levels exceeding 200 µg/ml. W. ugandensis has a much lower safety level below 50 µg/ml.

Except for *Warbugia ugandensis* which is clearly cytotoxic at IC_50_ of less than 50 *µg/ml,* the other medicinal plant extracts promoted the proliferation of IEC-6 cells normally at IC_50_ over 200 *µg/ml* (Figure 2). A concentration of 100 *µg/ml* and above is normally considered quite safe.

### Expression of GAPDH and IL 7 in IEC -6 Cells Exposed to Various Traditionally and Commonly Used Crude Medicinal Plant Extracts

**Figure 3:**
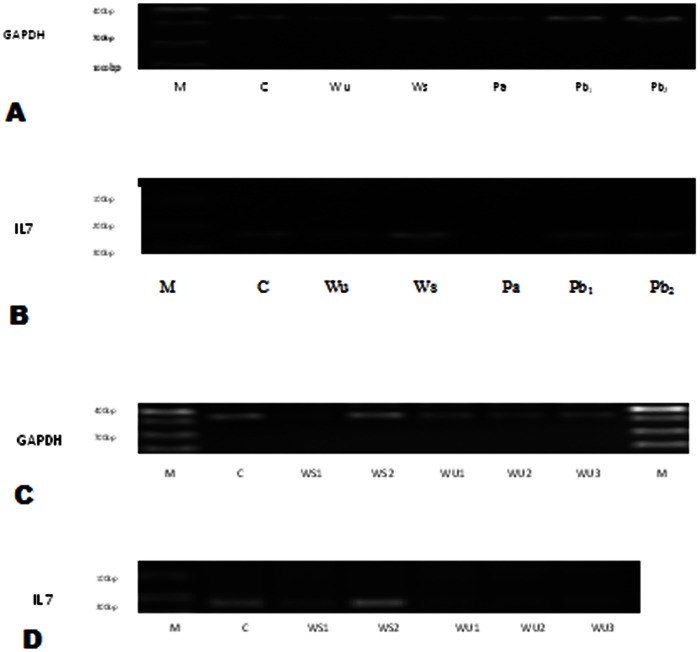
72 hr expression of GAPDH and IL-7 on treatment with different herbs. A&B represents GAPDH & IL 7 respectively at concentrations: WU 50 µg/ml, WS 100 µg/ml, PA 50 µg/ml, PB1 500 µg/ml & PB2 1000 µg/ml respectively. C&D represents GAPDH & IL 7 at concentrations: WU1 83.33 µg/ml, WU2 41.67 µg/ml, WU3 16.67 µg/ml, WS1 666.67 µg/ml & WS2 333.33 µg/ml respectively. IL 7 is not expressed in presence of P. africana. Doubling P. barbatus concentration does not affect expression in either direction. Abbreviations; M; marker, C; control, WU; Warbugia ugandensis, WS; Withania somnifera, PA; Prunus africana, PB; Plectrunthus barbatus.

**Figure 4:**
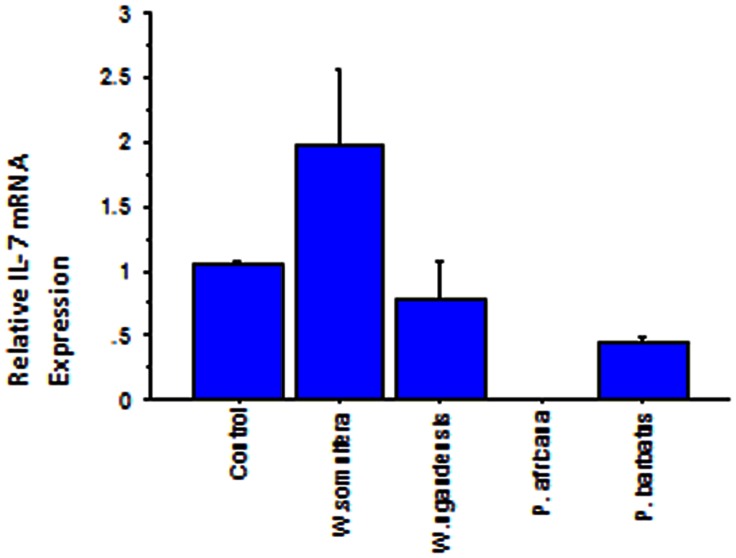
Bar chart depicting the relative IL 7 mRNA expression in IEC-6 cells subjected to the 4 medicinal plants. The assay was replicated 3 times. W. somnifera up-regulates IL-7 to approximately 2 times.

The Results represent expression of GAPDH and IL-7 in IEC-6 cells subjected to medicinal plant extracts at concentrations which do not inhibit normal cell growth (Figure 3 & 4, Table 3). GAPDH served as an internal control. The base line level for IL-7 expression in the control was one. *W. somnifera* (Ws) up regulates IL-7 expression to approximately two times; the optimum up regulation of IL 7 is achieved at a low concentration of 100 µg/ml (Figure 3 & 4). There is a dose-dependent response regarding GAPDH and IL7 expression when applying *W. somnifera* extracts. *W. ugandensis* (Wu) while being cytotoxic at higher doses down regulates IL-7 to 0.783 times in the presumably healthy growing IEC-6 cells at lower concentrations (Table 3, Figure 4). IL-7 was not expressed in the presence of *P. africana* (Pa) extract attributable to possible IL-7 gene silencing. *P. barbatus* (Pb) like *W. ugandensis* down regulates expression of IL-7 but in this case by almost half (0.451). Doubling the concentration of Pb did not reveal any additional effect on expression of the two genes and might indicate safety and lower optimum levels.

**Table 3:** Relative IL 7 mRNA expression in IEC-6 cells subjected to the 4 medicinal plants.

Medicinal plant Extract	Relative Mean IL-7 mRNA Expression±SD
Control	1.041±0.044
*Withania somnifera*	1.963±0.579*
*Warbugia ugandensis*	0.783±0.300
*Prunus africana*	0.000±0.000*
*Plectranthus barbatus*	0.451±0.032*

* Significantly different from the control at P≤0.05.

## Discussion

Most traditional medicinal plants in use today have no scientific data on their bioactivity and levels of safety or even how they are likely to affect each other when used as combinations in medicines. Furthermore scanty research has been done on their mechanisms of action considering that most are orally consumed. This study has demonstrated that *Withania somnifera, Warbugia ugandensis, Prunus africana* and *Plectrunthus barbatus* used in traditional medicine have both bactericidal and fungicidal activity (Table 1). Importance as traditional medicines cannot be overemphasized as they are not only widely used in Kenya but, worldwide [16], [17], [18]. They are claimed not just as antimicrobials but also as immunomodulators, among a host of other activities and they are used as such. In this study *W. ugandensis* has been shown to be cytotoxic with IC_50_<50 *µg/ml* when evaluated in IEC −6 cells. Never the less it has also been shown to have MIC values of less than 0.78 *mg/ml* in antifungal and antibacterial evaluations (Table 2). Bii et al. (2010) reported good activity of methanol extracts of *prunus africana* against bacterial and fungal strains [15]. Similarly, in this study, methanol extract of *P. africana* was found have good activity while the ethyl acetate fraction had moderate activity against *Staphylococcus aureus* and Methicilin Resistance *Staphylococcus aureus*. *W. somnifera, P. africana* and *P. barbatus* have IC_50_ cytoxicity levels much higher than 100 *µg/ml* when evaluated in IEC-6 cells. They can therefore be considered as relatively safe in traditional medicine justifying their continued use. *W. ugandensis* has wide use in Kenya as an antimicrobial agent however, caution should be excised when using it and when used only in small amounts. It most likely works through direct cytoxicity leading to inhibition of cell growth. Most traditional medicines are orally consumed. The intestinal epithelial cells come directly into contact with the plant medicines. IEC-6 cells express IL-7 a cytokine associated with immunopotentiation [24], [26]. *W. somnifera* has been shown in this study to up regulate IL-7 to two times. *W. somnifera* has been reported in treatment of cancer and various other diseases [19]. It can be deduced that this is the most likely mechanism by which it works. Research on animal cell cultures has shown that the herb decreases the levels of the nuclear factor kappaB, suppresses the intercellular tumor necrosis factor, and potentiates apoptotic signalling in cancerous cell lines [34]. *W. somnifera* too has been shown to have stimulatory effects, both in vitro and in vivo, on the generation of cytotoxic T lymphocytes, and a demonstrated potential to reduce tumor growth [35]. Our results agree with these claims as supported by the cidal and immunopotentiation effects.

**Figure 5:**
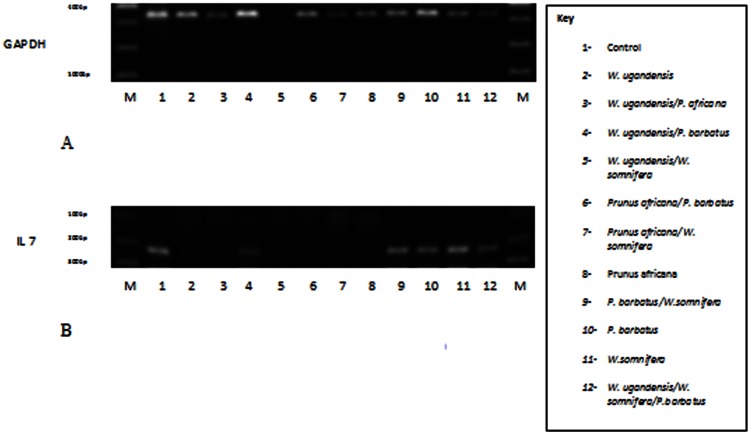
72 hr expression of GAPDH and IL 7 on treatment with different combinations of the 4 plant extracts. A & B represent GAPDH & IL 7 respectively. Combination ratios, WU/PA 1∶2, WU/PB 1∶4, WU/WS 1∶1, PA/PB 1∶2, PA/WS 2∶1, PB/WS 2∶1, PB/WS 4∶1, WU/WS/PB, 1∶1:4. P. africana was able to shut down the expression of IL 7 irrespective of the combination used.

Other medicinal plants considered in this study down regulate or completely shut down IL-7 expression therefore unlikely to work as immunomodulators. Combining *W. somnifera* with the other medicinal plants adversely affects its effectiveness as an immunomodulator. *Prunus africana* another widely used medicinal plant shuts down expression of IL 7 completely at tested concentrations. It is possible that this is the mechanism by which *P. africana* works in traditional medicine by silencing certain genes. However this theory should be pursued further. This effect is extended when used in combination with the other extracts (Figure 5A&B). Depending on desired results, care should be taken when using *P. africana* in combination treatment. *P. barbatus* although not evaluated as an antimicrobial in this study, the methanolic extract of *P. barbatus* has potent antibacterial activity against gram positive bacteria including *S. aureus* and antifungal effect against *C. albicans* [36], [37]. In this study, it down regulated IL 7 mRNA expression while, being relatively safe with IC_50_>200 µg/ml. It has wide usage in traditional medicine and its activity might be linked to its down regulating effects on the gene under investigation and possibly a host of many others. *W. somnifera* has the best antimicrobial (with a low figure of 1.5625 mg/ml against *Trichophyton mentagrophytes*), immunopotentiation (2 times IL 7 mRNA expression) and safety level (Table 1, Figure 2 & 4). A similar report has been made by Lakshmi-Chandra Mishra, *et al*., 2000 [19].

Flagellin reportedly down-regulates mRNA expression and secretion of IL-7 by IECs hence local lymphocyte pool may be regulated by the gut bacterial load, via control of IL-7 secretion [38]. The bactericidal effect of investigated medicinal plants could act directly on gut bacterial flora reducing the load. This may indirectly lead to up regulation of IL-7 which stimulates immune organs to produce and release more CD4+ and CD8+ lymphocytes raising the immune level leading to clearance of invading microbes.

### Conclusion

Use of *Withania somnifera, Warbugia ugandensis, Prunus africana* and *Plectrunthus barbatus* in traditional medicine has been justified. The probable mechanisms of action are bactericidal, fungicidal and immunopotentiation. Fractionation has yielded active fractions from *W. ugandensis* and *W. somnifera*. The bioassay-guided fractionation procedure to characterize and isolate the antibacterial and antifungal active constituents is in progress.
